# Functional annotation of structural ncRNAs within enhancer RNAs in the human genome: implications for human disease

**DOI:** 10.1038/s41598-017-15822-7

**Published:** 2017-11-14

**Authors:** Chao Ren, Feng Liu, Zhangyi Ouyang, Gaole An, Chenghui Zhao, Jun Shuai, Shuhong Cai, Xiaochen Bo, Wenjie Shu

**Affiliations:** 1 Department of Biotechnology, Beijing Institute of Radiation Medicine, Beijing, China; 2 Department of Information, The 188th Hospital of ChaoZhou, ChaoZhou, China

## Abstract

Enhancer RNAs (eRNAs) are a novel class of non-coding RNA (ncRNA) molecules transcribed from the DNA sequences of enhancer regions. Despite extensive efforts devoted to revealing the potential functions and underlying mechanisms of eRNAs, it remains an open question whether eRNAs are mere transcriptional noise or relevant biologically functional species. Here, we identified a catalogue of eRNAs in a broad range of human cell/tissue types and extended our understanding of eRNAs by demonstrating their multi-omic signatures. Gene Ontology (GO) analysis revealed that eRNAs play key roles in human cell identity. Furthermore, we detected numerous known and novel functional RNA structures within eRNA regions. To better characterize the *cis*-regulatory effects of non-coding variation in these structural ncRNAs, we performed a comprehensive analysis of the genetic variants of structural ncRNAs in eRNA regions that are associated with inflammatory autoimmune diseases. Disease-associated variants of the structural ncRNAs were disproportionately enriched in immune-specific cell types. We also identified riboSNitches in lymphoid eRNAs and investigated the potential pathogenic mechanisms by which eRNAs might function in autoimmune diseases. Collectively, our findings offer valuable insights into the function of eRNAs and suggest that eRNAs might be effective diagnostic and therapeutic targets for human diseases.

## Introduction

Enhancers are distal-acting DNA regulatory elements that can recruit transcription factors (TFs) and coactivators to up-regulate the transcription of their distal target genes by forming promoter-enhancer looping interactions^1^. Thus, enhancers play key roles in the precise spatiotemporal control of gene expression required for development, differentiation and homeostasis^2,3^. In 2010, two studies simultaneously presented genome-wide evidence of widespread RNA polymerase II (RNAPII) binding at non-coding enhancers which transcribed a novel class of enhancer RNAs (eRNAs)^1,4^. Later studies revealed the existence of two types of eRNAs (1D-eRNA and 2D-eRNA) distinguished by their size, polyadenylation state, and transcriptional directionality^5^. 1D-eRNAs are long (>4 kb), polyadenylated RNAs that undergo unidirectional transcription, whereas 2D-eRNAs are relatively short (<2 kb), are not polyadenylated, and undergo bidirectional transcription.

Since the discovery of eRNAs, extensive efforts have been devoted to revealing their potential functions and underlying mechanisms. Several studies have reported that eRNAs are a strong hallmark of active enhancers and that the transcription of eRNAs correlates with the activity of active enhancers^6–8^. An increasing number of studies support the idea that the eRNA transcription is not transcriptional noise but is responsible for biological functions and the regulation of transcriptional programmes^5,9,10^. However, whether the act of transcription or the transcripts *per se* convey the functionality remains open for debate. Some studies have found that the act of transcription outweighs the importance of the eRNA transcripts^11^ and that the inhibition of eRNA transcription does not affect enhancer-promoter looping with 3 C^12^, whereas an increasing number of studies have presented evidence that eRNA transcripts are important for proper enhancer-promoter looping and that the eRNA transcripts themselves play a functional role in regulating the transcription of target genes^13–17^.

Nonetheless, it is clear that not all enhancers are transcribed at the same time and that the active enhancers transcribing eRNAs may represent only a small fraction of all enhancers^1,2,18–20^. The differential transcription of active enhancers across cell types and tissues helps explain the diversity of cell types and tissues sharing the same genome. However, the existing knowledge of eRNAs is greatly inadequate, and the mechanisms of eRNA activity remain a mystery. Therefore, it is of interest to investigate the differences in eRNA transcription and the function of eRNAs across cell types and tissues. Furthermore, regulatory RNA structures play an important role in gene regulation and function, and many novel structured RNAs have been identified^21–24^. In addition, it has been reported that genetic variation can induce changes in RNA structure^25–28^ and that variation in enhancers is closely associated with human diseases^2,29–31^. Thus, investigating eRNA transcription from a structural perspective should help identify and validate structural RNA elements that are involved in diverse cellular processes and thereby increase our understanding of eRNA function.

In this study, we created a catalogue of eRNA regions using genome-wide chromatin immunoprecipitation-sequencing (ChIP-seq) and RNA sequencing (RNA-seq) data across 50 human cell and tissue types. We characterized these eRNA regions and extended our understanding of their functionality by analysing their multi-omic signatures, including genomic, epigenetic, transcriptomic, and chromatin interaction characteristics. Gene Ontology (GO) analysis revealed that eRNA regions are associated with genes that control and define cell identity. Furthermore, we detected and identified numerous known and novel functional RNA structures within eRNA regions. To better characterize the *cis*-regulatory effects of non-coding variation, we performed a comprehensive association analysis between the structural ncRNAs in eRNA regions and genetic variants associated with inflammatory autoimmune diseases. We observed that these disease-associated variants are disproportionately enriched in structural ncRNAs. Importantly, this enrichment is biased towards immune-specific cell types. Furthermore, we detected riboSNitches in lymphoid eRNA regions and investigated the potential pathogenic mechanisms by which eRNAs can function in autoimmune diseases. Collectively, our findings offer valuable insights into the function of eRNAs and reveal their potential as effective diagnostic and therapeutic targets for human diseases.

## Results

### Identification and characterization of enhancer RNAs

We identified enhancers by an “*ab initio*” assembly pipeline using histone modifications and GENCODE (V19)^32^ annotation, and we identified 2,373 intergenic candidate enhancers in human embryonic stem cells (ESCs) (Fig. S1, Materials and Methods). To validate the identified candidate enhancers, we profiled 12 multi-omic signatures in these candidate enhancer regions in H1-hESCs, including 2 TFs, 1 cofactor, 6 histone modifications, RNAPII, DNase I-hypersensitive sites (DHSs), and DNA methylation (Figs S2 and S3). H3K4me3, P300, Nanog, Pou5f1, and Pol2 were enriched in enhancers, whereas H3K4me3, DNA methylation, and H3K36me3 were depleted in enhancers. The profile patterns of these signatures were consistent with the profiles presented in previous studies^1,5,33^ (Fig. S3). Furthermore, we compared the various multi-omic signature profiles of the candidate enhancers with the profiles of promoters, long intergenic non-coding RNAs (lincRNA) genes, and ribosomal RNA (rRNA) genes. The candidate enhancers were markedly different from these regulatory elements (Fig. S3). Enhancers have a high H3K4me1 to H3K4me3 ratio and are bound by unique transcription factors (Nanog and Pou5f1 for H1-hESCs) and the coactivator P300. Promoters have a low H3K4me1 to H3K4me3 ratio and are bound by RNA polymerase II with their downstream protein-coding genes occupied by H3K36me3 and H3K27me3. Compared to enhancers and promoters, the epigenetic and transcriptional features in lincRNA genes and rRNA genes are not obvious. Together, these results indicated that the candidate enhancers we identified were reliable and accurate.

To discriminate between enhancers that are classified into eRNA regions and weakly-transcribed enhancers, we used high-throughput mRNA-seq data to count the RNA-seq tags at a given candidate enhancer (Materials and Methods). We detected polyadenylated 1D-eRNA regions in 837 of the 2,373 (35.3%) intergenic candidate enhancers in H1-hESCs. Then, we investigated the size, distance to the nearest transcription start site (TSS), evolutionary conservation, and chromatin 3D structure of both the eRNA regions and the weakly-transcribed enhancers (Fig. S4). Relative to the weakly-transcribed enhancers, eRNA regions were longer, closer to the nearest TSS, less evolutionarily conserved, and had looser spatial structures.

**Figure 1:**
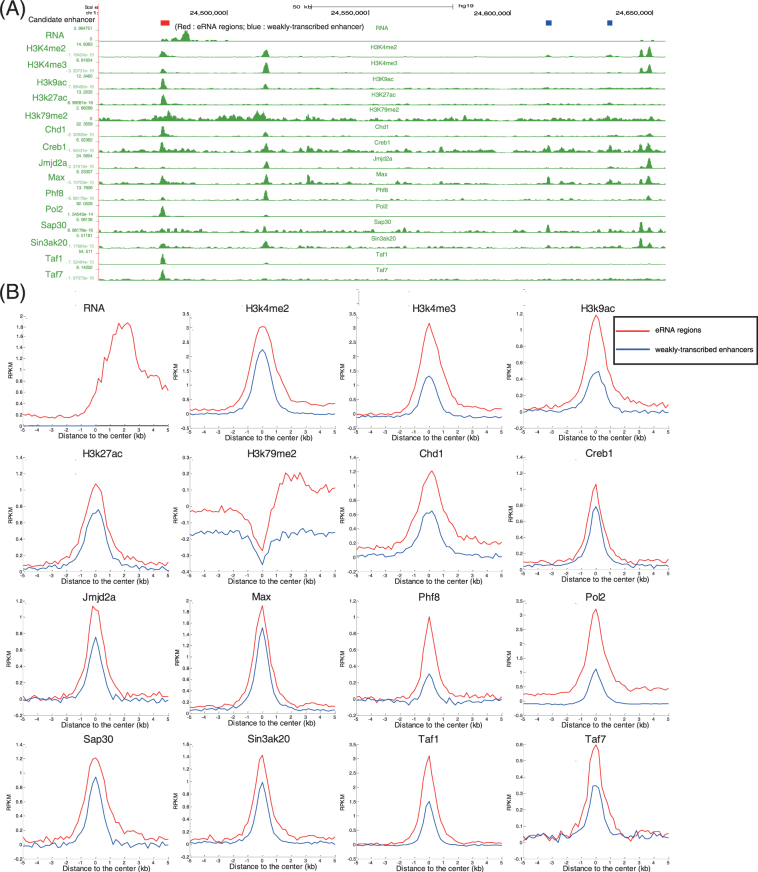
Identification and characterization of the eRNA regions in hESCs. (**A**) UCSC browser display of epigenetic signatures, transcription factors, coactivators, and transcription status for a representative locus in chr1: 24,455,067-24,654,494. The red rectangle indicates an eRNA region, and blue rectangles indicate weakly-transcribed regions. (**B**) Metagene representations of the mean multi-omic signature signal for DNase I, RNAPII, histone modifications, RNA-seq, and chromatin regulators across eRNA regions (red) and weakly-transcribed enhancers (blue) regions. Metagenes are centred on the eRNA regions (5,863 bp and 11,890 bp for weakly-transcribed enhancers and enhancer RNA regions, respectively) with 5 kb on both sides of each enhancer region.

To identify additional characteristics distinguishing eRNA regions from weakly-transcribed enhancers, we investigated how the 16 compiled multi-omic signatures in H1-hESCs are associated with eRNA regions and weakly-transcribed enhancers across the ESC genome (Fig. 1A). We found that these signatures were enriched in eRNA regions relative to weakly-transcribed enhancers (Fig. 1B). Noticeably, these enriched signatures were all involved in transcription regulation. For example, RNAPII can catalyse the transcription of DNA to synthesize RNA^1,5,30,33^; H3K4me2 and H3K4me3 play important roles in the positive regulation of transcription^34–36^; transcription initiation factor TFIID subunit 1 (Taf1) is the largest component and the core scaffold of the TFIID basal TF complex and can be recruited to tissue-specific enhancers^33^; and chromodomain-helicase-DNA-binding protein 1 (Chd1) is an ATP-dependent chromatin-remodelling factor that can regulate RNAPII transcription and that plays key roles in maintaining open chromatin and pluripotency in ESCs^37,38^. In addition, we investigated the enrichment of TF binding motifs in eRNA regions (Tables S1 and S2). The binding motifs, which mainly play key roles in regulated programmes of gene transcription, showed significant enrichment in eRNA regions relative to background expectations. Furthermore, eRNA regions were enriched for these motifs compared with weakly-transcribed enhancers (Table S3). Taken together, the widespread enrichment of the signatures and TF binding motifs at eRNA regions suggested that eRNAs might participate in many regulated programmes of gene transcription.

### Identification of eRNA regions in many cell types and tissues

**Figure 2:**
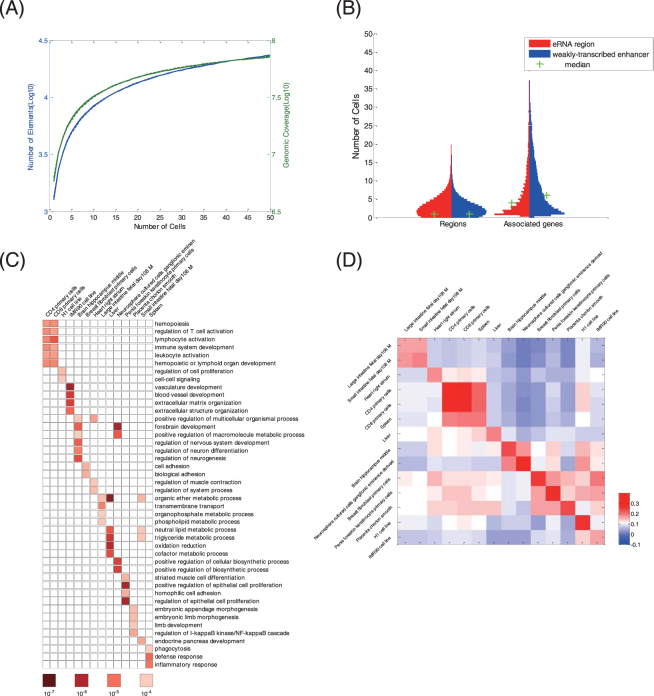
eRNAs in many cell types. (**A**) The saturation curves of eRNA regions obtained by Weibull fitting. Mean eRNA region count (blue line) and mean genome coverage (green line) for *x* cell types after clustering from 20,000 random samples (solid line) were fit using the Weibull distribution (corresponding dashed line). The elements are non-overlapping and have a maximum length of 5,000 bp. (**B**) Cell specificity of eRNA regions (red) and weakly-transcribed enhancers (blue) regions and their associated genes displayed as a violin plot. Medians are marked with green crosses. (**C**) Gene Ontology terms for eRNA-associated genes in 14 human cell/tissue types with corresponding *P*-values. (**D**) Heatmap showing the correlations between the expression levels of eRNAs and their associated genes across 14 human cell/tissue types. Colour scale reflects the density of Spearman correlation coefficients.

To further explore the function of eRNAs, it would be useful to identify these elements and their associated genes in as many human cell types and tissues as possible. To this end, we used ChIP-seq data and mRNA-seq data on H3K27ac, H3K4me1, and H3K4me3 to generate a catalogue of eRNA regions across 50 different human cell and tissue types in the Roadmap Epigenomics Project^39^ using the aforementioned workflow in H1-hESCs. In total, we identified 23,878 eRNA regions, which cover 55.2 Mb (1.8%) of the human genome. To assess the rate of discovery of new eRNA regions, we performed a saturation analysis as described in our recent study^40^ and predicted saturation at a count of approximately 37,222 eRNA regions (standard deviation = 5,309) and coverage of approximately 90.0 Mb (standard deviation = 3.5 Mb) (Fig. 2A). Our results suggest that we have found more than 60% of the total estimated eRNA regions.

A substantial portion of these eRNA regions were typically much more cell selective than weakly-transcribed enhancers (Fig. 2B); the average eRNA region was detected in 7 cell types, whereas the average weakly-transcribed enhancer was detected in 21 cell types. In addition, genes associated with eRNA regions were largely cell selective, whereas genes associated with weakly-transcribed enhancers typically exhibited higher prevalence across cell types (Fig. 2B). GO analysis of genes associated with eRNA regions revealed that they are actively engaged in promoting the mRNA transcription of their respective cell/tissue types (Fig. 2C). For example, eRNA regions in lymphoid cells participate in processes such as the regulation of T cell activation, lymphocyte activation, and immune system development; eRNA regions in H1 cells are major participants in the regulation of cell proliferation; eRNA regions in liver tissue function in the metabolic process; and eRNA regions in spleen tissue play roles in the defence response and inflammatory response.

To investigate whether the activity levels of eRNAs correlate with the mRNA levels of nearby genes, we used RNA-seq data and calculated their expression levels (Fig. S5). We found that the eRNA expression levels are strongly correlated with the mRNA expression levels at nearby genes (Fig. 2D). Moreover, this correlation demonstrates cell type-specific behaviour and is higher than the correlation between weakly-transcribed enhancers and their associated genes. The positive correlation between the expression levels of eRNA and mRNA suggests that eRNA synthesis may occur specifically at enhancers that are actively engaged in promoting mRNA synthesis.

To further validate our identified enhancers and eRNA regions, we compared them with regions identified in previous studies. The Roadmap Epigenome Consortium defined 2,328,936 putative enhancer regions across 127 cell/tissue types with ChromHMM^41^. They provided the most comprehensive map of the human epigenome landscape. We compared our identified enhancers with their comprehensive enhancer sets in 11 common cell/tissue types. On average, 76.0% of our enhancers overlapped with those defined by the Roadmap Epigenome Consortium in the corresponding cell/tissue (Table S4). We profiled the multi-omic signatures, including TFs and coactivator, histone modifications, RNAPII, and DHSs, and found that these signatures were enriched in our enhancer regions relative to enhancers defined by the Roadmap Epigenome Consortium (Fig. S6). Hon *et al*. defined e-lncRNA by selecting reliable lncRNA (long non-coding RNA) overlapping with enhancer regions^42^. Compared to these e-lncRNAs, our eRNAs demonstrate higher enhancer and transcriptional activity based on the different multi-omic signatures, including 2 TFs, 1 cofactor, 3 histone modifications, DHSs, and RNAPII (Fig. S7). Together, we archived more stringent and reliable enhancer regions and eRNA regions.

### Identification of known structural ncRNAs

To further extend our understanding of eRNA function, we investigated eRNA transcripts from a structural perspective and explored the consensus RNA secondary structure profiles within eRNA regions. We used the Infernal software package^43,44^ and 2,450 covariance models (CMs) from the Rfam database (V12.0)^45^ to search for known structural ncRNAs in eRNA regions across 10 human lymphoid cell types (Materials and Methods). The enhancers and ncRNAs that control lymphoid cell states are likely to be understood better than those in other cell types, making lymphoid commitment an excellent model for identifying structural ncRNA components^46–49^.

**Figure 3:**
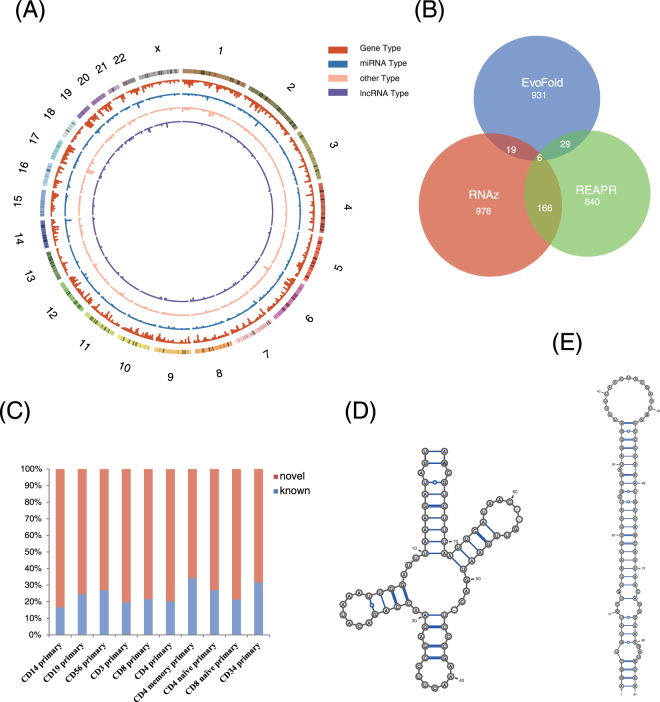
Repertoire of structural ncRNAs in lymphoid eRNA regions. (**A**) Genomic distribution of ncRNAs discovered by known structural ncRNA models. A master list of ncRNAs discovered across 10 human lymphoid cell types was created. The results were categorized based on CM types provided by the Rfam database (V12.0). (**B**) Venn diagrams of novel structural ncRNAs identified by RNAz, REAPR, and EvoFold in CD4 primary cells. (**C**) Percentage of novel structural ncRNAs confirmed by comparison to Rfam database using BLASTN across 10 human lymphoid cell types. (**D**) Novel structural ncRNA similar to tRNA. (**E**) Novel structural ncRNA similar to miRNA miR-155.

In total, we detected 18,767 unique structural ncRNA hits in the 410 CMs (16.7% of the 2,450) in lymphoid eRNA regions (Fig. 3A). These hits contained a large number and variety of functional ncRNAs. We identified approximately 30% (161/530) of miRNAs, 36% (78/216) of lncRNAs, 100% (2/2) of tRNAs, and 67% (2/3) of snRNAs within these lymphoid eRNA regions. Among the identified structural ncRNAs, we detected a substantial enrichment of metazoan_SRP (metazoan signal recognition particle RNA), which is a component of signal recognition particle RNA that plays important roles in both co-translational translocation and post-translational transport^50^ (Fig. 3A). Other substantially enriched structural ncRNAs included SCARNA7, tRNA, and miR-548, which are all reported to be functional ncRNAs in human lymphoid cells^51–53^. Overall, we identified a large number and variety of functional ncRNAs with known secondary structure in lymphoid eRNA regions.

### Detection of novel structural ncRNAs

Furthermore, we employed three different popular methods, RNAz^54^, REAPR^22^, and EvoFold^55^, to identify novel structural ncRNAs in lymphoid eRNA regions (Materials and Methods). We selected the predictions identified by at least two methods as our results (Fig. 3B, an example from CD4 primary cells). On average, we identified 116 structural ncRNAs in the eRNA regions of 10 human lymphoid cell types, ranging from 52 in CD4 naïve primary cells to 221 in CD3 primary cells. Comparing these results with the Rfam database using BLASTN^56^, we found that on average, 75.7% of these structural ncRNAs are novel (Fig. 3C), and the most common structure in the novel ncRNAs is the stem-loop structure.

**Figure 4:**
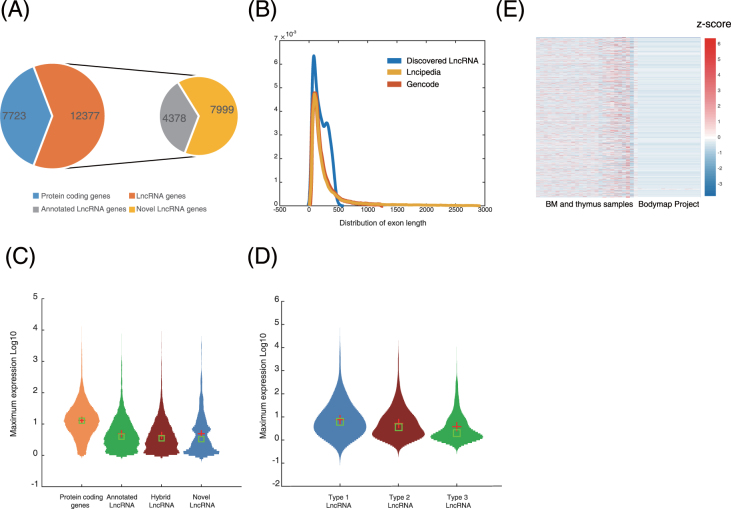
Identification and characterization of lncRNAs in lymphoid eRNA regions (**A**) Quantification of expressed protein-coding genes, lncRNA genes, annotated lncRNA genes (in GENCODE V19 or LNCipedia database), and novel lncRNA genes (not annotated in these databases). (**B**) Size distribution of novel lncRNA transcripts. Data for transcripts from protein-coding databases and annotated lncRNA databases are depicted for comparison. (**C**) Expression of protein-coding genes, annotated and novel lncRNA genes (previously defined), and hybrid lncRNA genes (the subset of annotated lncRNA genes for which we discovered previously unknown transcripts) presented as the maximum FPKM values (of 20 samples). Red crosses indicate the mean, green squares indicate the median, and the outline indicates range. (**D**) Violin plot of transcript expression levels. For each transcript, the maximum expression value was used for the plot. Type 1, novel transcripts from novel loci or annotated loci; Type 2, transcripts from annotated loci that have novel isoforms; Type 3, annotated transcripts from known loci. Expression levels were higher for novel lncRNA transcripts than for annotated lncRNA transcripts. (**E**) Expression of newly identified lncRNA genes in BM and thymus samples (left) and 10 samples from the Human BodyMap project (right; Table S5).

To further reveal the potential functions of the novel structural ncRNAs, we used NoFold^57^ to cluster these novel structural ncRNAs with all the known structural ncRNAs from the Rfam database in an Rfam-based feature space. Based on the high importance of secondary structure for functional ncRNA, we inferred the functions of the novel structural ncRNAs from the functions of the clustered known structural ncRNAs. A novel structural ncRNA identified in the eRNA regions of CD4 primary cells has a similar structure to the cloverleaf of tRNA, which plays key roles in human lymphoid cells^52^ (Fig. 4D). This similarity suggested that the novel structural ncRNA might have a similar function to that of tRNA. In addition, we identified another novel structural ncRNA in the eRNA regions of CD4 primary cells whose structure was similar to the stem-loop structure of miR-155 (Fig. 4E). miR-155 is one of five miRNAs (miR-142, miR-144, miR-150, miR-155, and miR-223) that are specific for haematopoietic cells, including B-cells, T-cells, monocytes, and granulocytes, and they have been reported to regulate the response of CD cells^58,59^. The similarity of the stem-loop structure between the newly identified structural ncRNA and miR-155 suggested that these RNAs might perform similar functions in CD cells.

### Identification of lncRNAs

To elucidate the lncRNA “landscape” of human lymphoid commitment, we used an “*ab initio*” assembly pipeline from a previous study^46^ to annotate an lncRNA catalogue from the 10 human lymphoid cell types (Materials and Methods). These lncRNA transcripts, which were confirmed by both CPAT^60^ and CPC^61^ analyses, were devoid of protein-coding potential. This comprehensive annotation covered a total of 22,339 lncRNA genes, of which 4,378 (19.6%) novel lncRNA genes are not annotated in existing lncRNA databases, including GENCODE^32^ (V19) and LNCipedia^62^ (V2.1) (Fig. 4A). Because each gene produces multiple transcripts by alternative splicing, 36,575 lncRNA transcripts were identified, and of these, 6,482 (17.7%) were previously unknown lncRNA transcripts (Table S6). When mapping these transcripts to lymphoid eRNA regions, we obtained 3,230 lncRNA genes and 3,329 lncRNA transcripts located within lymphoid eRNA regions, including 529 previously unannotated lncRNA genes and 645 previously unknown lncRNA transcripts (Table S6).

We then estimated the expression levels for lncRNA genes in the final lncRNA annotation. We found that a total of 10,531 lncRNA genes, of which 2,145 were previously unknown, were expressed at FPKM (Fragments Per Kilobase of exon model per Million mapped reads) values > 1 in at least one sample (Fig. 4A). The size distribution of the newly identified lncRNA transcripts was similar to that of previously annotated lncRNAs^32,62^ (Fig. 4B). Notably, the newly identified lncRNA transcripts, regardless of whether they were transcribed from previously unannotated loci or annotated loci, showed more abundant expression than previously annotated lncRNAs in these haematopoietic populations (Fig. 4C,D). Analysis of RNA-seq data from the Human BodyMap project (Table S5) revealed that the newly identified lncRNA genes from our data were highly specific to the human lymphoid cells we studied (Fig. 4E).

### Detection of riboSNitches

Single nucleotide polymorphisms (SNPs) and other mutations can alter RNA structure, affect its molecular function, and thereby cause phenotypic effects. The accurate prediction of riboSNitches, which are RNAs with large structural disparities caused by a single nucleotide variant (SNV)^25,63,64^, is crucial to interpreting RNA structures as they pertain to individual phenotypes. Thus, we collected a list of 68,295 SNPs from the NHGRI-EBI GWAS catalogue^65^ and the ClinVar database^66^ and compiled a list of 504 SNPs related to autoimmune disease based on annotations and literature reports. In addition, we generated a list of SNPs in strong linkage disequilibrium (LD) with autoimmune disease-associated SNPs using the LD cutoff of *r* ^2^ > 0.8. Of these 504 SNPs, 204 (40.6%) occurred in 197 structural ncRNA loci in lymphoid eRNA regions. Moreover, 17,002 (39.5%) were in strong LD (*r* ^2^ > 0.8) with these 504 SNPs in 9,324 structural ncRNA loci in lymphoid eRNA regions. In total, 17,506 SNPs were defined as autoimmune disease-associated SNPs. Notably, these autoimmune disease-associated SNPs, regardless of whether they were annotated or in strong LD with annotated SNPs, showed a greater disproportional enrichment in these structural ncRNA loci than in random structural ncRNA loci (permutation test, *P* < 10^−4^).

To analyse the structural ncRNAs in which these autoimmune disease-associated SNPs resided, we used RNAsnp^26^ with the default parameters to predict the effects of SNPs on local RNA secondary structure (Materials and Methods). We found that lymphoid structural ncRNA loci were more likely than random loci to be disrupted by SNPs (Fig. S8A). Additionally, using RNAsnp with *P* < 0.2, we defined riboSNitches as RNAs that undergo large structural changes when disrupted by autoimmune disease-associated SNPs. In total, 1,764 of these structural ncRNAs in lymphoid eRNA regions were identified as riboSNitches. Among them, 23 were directly associated with GWAS or ClinVar SNPs, whereas 1,741 were associated with SNPs in strong LD with GWAS or ClinVar SNPs. Importantly, the newly identified riboSNitches, regardless of whether they were in direct association with GWAS or ClinVar SNPs or in strong LD with GWAS or ClinVar SNPs, were disproportionately enriched in these lymphoid structural ncRNA loci relative to random structural ncRNA loci (permutation test, *P* < 10^−4^).

### Examples of riboSNitches

**Figure 5:**
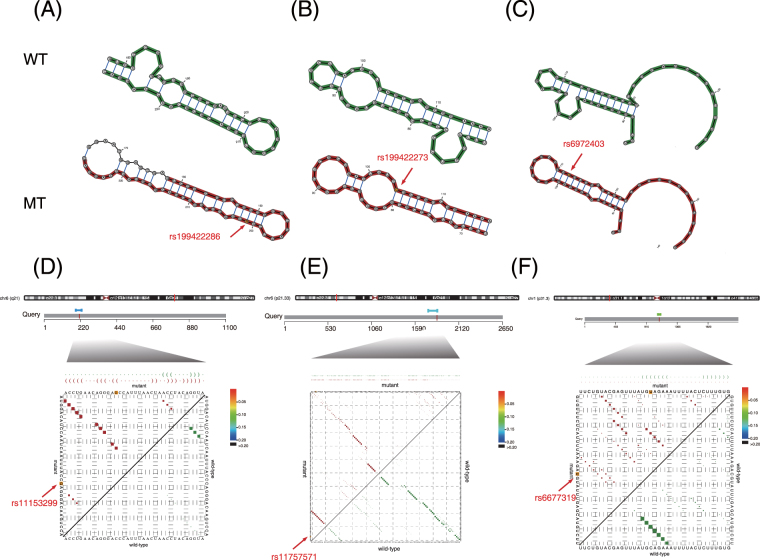
Detection of riboSNitches in lymphoid eRNA regions. (**A**) The partial effect of SNP rs19942286 on TERC in lymphoid eRNA regions. (**B**) The partial effect of SNP rs19942273 on TERC in lymphoid eRNA regions. (**C**) The partial effect of SNP rs6972403 on Hsp83_3_UTR in lymphoid eRNA regions. (**D**) The partial effect of SNP rs11153299 on TRAF3IP2-AS1 in lymphoid eRNA regions. (**E**) The partial effect of SNP rs11757571 on lnc-MICB-3:1 in lymphoid eRNA regions. (**F**) The partial effect of SNP rs6677319 on lnc-IL12RB2-1 in lymphoid eRNA regions.

To gain further insight into the relationship between SNPs and structural ncRNAs, especially riboSNitches, we focused on several structural ncRNAs that underwent large structural changes when disrupted by autoimmune disease-associated SNPs (Fig. 5).

Mutations in the telomerase RNA component (TERC), an RNA component of telomerase and the template for telomere replication by telomerase, can cause dyskeratosis congenita (DC) and might also be associated with some cases of aplastic anaemia (AA). DC is a disorder that is related to congenital bone marrow disease, whereas AA is a rare disease in which the bone marrow and the haematopoietic stem cells that reside there are damaged. Three SNPs (rs199422286, rs199422260, and rs199422273) were found in a locus of the CM “Telomerase-vert” that was located within TERC in lymphoid eRNA regions, and they had a significant effect on the structure of TERC (Figs 5A,B and S8B). SNPs rs19942286 and rs19942260 are related to DC^67,68^, whereas SNP rs19942273 is related to AA, in which the pathway “Telomere Extension by Telomerase” plays an important role^68^.

Although only a few GWAS or ClinVar SNPs have significant local structural effects, many risk SNPs, i.e., SNPs in strong LD (*r* ^2^ > 0.8) with GWAS or ClinVar SNPs, have significant effects on structural ncRNAs within lymphoid eRNA regions. SNP rs6972403, which is in strong LD with SNP rs4722404, fell into a locus of the heat shock protein 83 3′ UTR (Hsp83_3_UTR) structural ncRNA (Fig. 5C). The heat shock protein family plays an important role in immunity^69^, and SNP rs4722404 has been reported to be strongly associated with the risk of atopic dermatitis (AD)^70,71^, which is a chronically relapsing inflammatory allergic response occurring in the skin that causes itching and flaking. We found that SNP rs6972403 disrupted the structure of Hsp83_3_UTR and caused a large structural disparity (Fig. 5C). SNPs rs11153299 and rs2038013 are in strong LD with GWAS SNP rs3851228, which is associated with inflammatory bowel disease (IBD)^72^. Both rs11153299 and rs2038013 are in the exon regions of the antisense RNA TRAF3IP2-AS1, which has been reported to have a relationship with IBD, and significantly disrupted its structure^72,73^ (Figs 5D and S8C). Together, these examples indicated that autoimmune disease-associated SNPs, whether located within structural ncRNAs or in strong LD with SNPs within structural ncRNAs, have great effects on the structural ncRNAs of autoimmune disease-related cells. Thus, they could cause dysfunction of these structural ncRNAs and might contribute to the corresponding autoimmune disease.

Furthermore, we found that SNPs associated with specific autoimmune diseases tend to cause large structural changes in the structural ncRNAs of disease-relevant cell types. These structural ncRNAs show *cis*-regulatory effects on nearby genes and influence the risk of specific autoimmune diseases. SNP rs11757571, in the exon region of lncRNA lnc-MICB-3:1, is in strong LD with the GWAS SNP rs9266406 (Fig. 5E), which is related to Behcet’s disease^74^, another well-known autoimmune disease involving inflammation of the blood vessels. The lncRNA lnc-MICB-3:1 is associated with the MICB (MHC Class I Polypeptide-Related Sequence B) gene, whose reduced expression has also recently been demonstrated to influence the risk of Behcet’s disease^75,76^. SNP rs6677319, which occurs in the lncRNA lnc-IL12RB2-1 (Fig. 5F), is in strong LD with GWAS SNPs rs11465804 and rs11209026^77^. Both SNPs rs11465804 and rs11209026 are reported to be associated with Crohn’s disease, another well-known autoimmune type of IBD. The lncRNA lnc-IL12RB2-1 is associated with the IL12RB2 (interleukin 12 receptor, beta 2) gene, the up-regulation of which has recently been demonstrated to influence the risk of Crohn’s disease^78,79^. These two SNPs (rs11757571 and rs6677319) and an additional two SNPs (rs11950065 and rs11762252) (Fig. S8D,E) can cause large structural disparities in structural ncRNAs.

**Figure 6:**
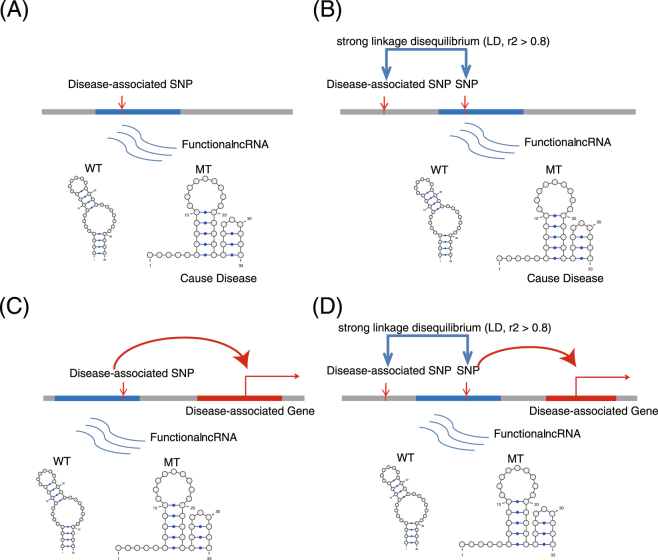
Different types of ncRNA disruption caused by SNPs. (**A**) The secondary structure of a functional ncRNA was directly disrupted by a disease-associated SNP in its sequence. (**B**) The secondary structure of a functional ncRNA was disrupted by a risk SNP in its sequence. (**C**) The *cis*-regulatory effect of an ncRNA on a nearby gene was disrupted due to the change in secondary structure caused by a disease-associated SNP. (**D**) The *cis*-regulatory effect of an ncRNA on a nearby gene was disrupted due to the change in secondary structure caused by a risk SNP.

Taken together, we found that autoimmune disease-associated SNPs, whether they were GWAS or ClinVar SNPs or in strong LD with GWAS or ClinVar SNPs, could significantly disrupt the structure of many ncRNAs in lymphoid eRNA regions. These examples of riboSNitches suggested that the significant impact of disease-associated SNPs on structural ncRNAs plays an important role in the function of the structural ncRNAs or their associated genes in disease-relevant cells. The large structural disparities caused by these SNPs might contribute to autoimmune-related diseases (Fig. 6).

## Discussion and Conclusions

Recent studies have provided increasing evidence of a direct role for eRNAs in enhancer function^80^. eRNAs regulate enhancer-promoter looping through the recruitment of cohesin^13,17^, direct chromatin accessibility^16^, assistance in recruiting mediators to promoters^81^, the facilitation of paused RNAPII transition into productive elongation^82^, and the stimulation of histone acetylation and transcription by binding directly to CBP^83^. Consistently, the knockdown of eRNAs affects the transcription of enhancer-associated genes^1,13,15,16,81^. Although extensive efforts have been devoted to revealing the functions and underlying mechanisms of eRNAs, the debate on these questions continues.

In this study, we first identified candidate enhancers by an “*ab initio*” assembly pipeline using three histone modifications and classified them into eRNA regions and weakly-transcribed enhancers. Then, we characterized the eRNA regions in ESCs and demonstrated that the eRNA regions show higher activity than weakly-transcribed enhancers. Further exploration of the enrichment of the multi-omic signatures and TF binding motifs at eRNA regions suggested that eRNAs may participate in various regulated gene transcription programmes.

Next, we created a catalogue of 23,878 eRNA regions covering 55.2 Mb (1.8%) of the human genome across 50 different human cell/tissue types. Saturation analysis suggests that we identified more than 60% of the total estimated eRNA regions. Both eRNA regions and their associated genes demonstrated heightened cell selectivity. Further GO analysis revealed that eRNAs play key roles in promoting gene transcription in the respective cell/tissue type. Moreover, the level of eRNA expression was positively correlated with the level of mRNA synthesis at nearby genes in a cell type-specific manner. Our results further confirmed previous findings that eRNA synthesis occurs specifically at enhancers that are actively engaged in promoting mRNA synthesis^1^.

Third, we explored the function of eRNAs by detecting known and novel functional RNA structures within these domains across 10 human lymphoid cell types. We discovered 18,767 unique structural ncRNAs with known secondary structure in lymphoid eRNA regions, which are a substantial portion of the functional ncRNAs provided by the Rfam database (V12.0). Additionally, we identified 1,158 structural ncRNAs in lymphoid eRNA regions, and 75.7% of these structural ncRNAs were novel. Furthermore, we identified 3,230 lncRNA genes and 3,239 lncRNA transcripts located within lymphoid eRNA regions, with 529 (16.4%) novel lncRNA genes and 645 (20.0%) novel lncRNA transcripts that were previously unannotated in existing lncRNA databases. Taken together, we identified a large number and variety of known and novel functional ncRNAs in lymphoid eRNA regions.

Finally, we found that genetic variants associated with a broad spectrum of inflammatory autoimmune diseases were disproportionately enriched in the structural ncRNAs in the eRNA regions of autoimmune disease-related cell types. To interpret the structural ncRNAs in which these autoimmune disease-associated SNPs resided, we predicted the effects of SNPs on the local RNA secondary structure and identified 1,764 of these structural ncRNAs within lymphoid eRNA regions as riboSNitches. Further examples of riboSNitches suggested that the significant impact of the autoimmune disease-associated SNPs on the structural ncRNAs plays an important role in the function of the structural ncRNAs or their associated genes in disease-related cells. The large structural disparities caused by these SNPs might contribute to these diseases.

Collectively, our eRNA catalogue provides a valuable resource for further study of the functions and underlying mechanisms of eRNAs. Our findings offer new insights into the functional roles of eRNAs in promoting gene transcription. The novel riboSNitches detected among the diverse structural ncRNAs within eRNA regions provide potentially effective diagnostic and therapeutic targets for human diseases. Thus, eRNAs play key roles in human cell identity and disease.

## Materials and Methods

### Datasets

All raw ChIP-seq data for histone modifications (H3K4me1, H3K4me3, and H3K27ac) and raw RNA-seq data across 50 cell/tissue types were downloaded from the Roadmap Epigenomics Project^39^. TFs, cofactors, histone modifications, DHSs, and DNA methylation in H1-hESCs were collected from the ENCODE Project^84^. Gene annotations were obtained from GENCODE (V19)^32^, and ncRNA annotations were obtained from NONCODE (http://noncode.org/)^85^, LNCipedia^62^, and GENCODE (V19). RNA-seq data collected from the Human BodyMap project are listed in Table S5.

### Data processing

We used Bowtie2^86^ and TopHat2^87^ to map the raw ChIP-seq data on histone modifications (H3K4me1, H3K4me3, and H3K27ac) and the RNA-seq data, respectively, to the human reference genome NCBI37/hg19. We employed the “callpeak” function of MACS2^88^ to call peaks of H3K4me1 and H3K27ac, and the *P*-values of H3K4me1 and H3K27ac were set to 10^−6^ and 10^−9^, respectively. The control for peak calling was obtained by merging all the replicates of the input using BEDOPS^89^ with the parameter “-everything”. For peaks of H3K4me1 and H3K27ac with two or more replicates, we chose only those that appeared in over 70% of the replicates and merged all the peaks from multiple replicates into a single peak file.

### Identification of eRNA regions

We first set up the workflow to locate candidate enhancers using histone modifications and gene annotation from GENCODE (V19)^32^ based on the method used by Kim *et al*.^1^ (Fig. S1). We began with the set of H3K27ac peaks after peak detection and replicate combination in the previous step. To be considered as enhancers, individual H3K27ac peaks had to satisfy the following criteria. (1) The H3K27ac peak had to be at least 1 kb away from all annotated TSSs in GENCODE (V19). (2) We excluded all H3K27ac peaks with a 5′-sequenced EST from the UCSC Genome Browser spliced EST track that has a 5′ end within 2 kb of the H3K27ac peak and that spans an annotated TSS. (3) Considering that active enhancers have a relatively high H3K4me1 levels and low H3K4me3 level^90^, we excluded H3K27ac peaks with abnormally high levels of both H3K4me1 and H3K4me3 enrichment in a 2-kb bin centred on the peak. We calculated the z-scores of H3K4me1 and H3K4me3 enrichment, which follow standard normal distributions. H3K4me1 and H3K4me3 enrichment with z-scores larger than 5 was defined as abnormally high according to the five sigma criterion. (4) The enrichment of H3K4me3 within a 2-kb window centred on the peak had to have a *z*-score less than 3. (5) An H3K4me1 peak had to be present within 2 kb of the H3K27ac peak. (6) We included intergenic H3K27ac peaks. (7) We excluded H3K27ac peaks that overlapped rRNA genes annotated by GENCODE (V19). Fig. S1 indicates the number of H3K27ac peaks of H1-hESCs at each stage of this filtering. In total, we identified 2,373 intergenic candidate enhancers in H1-hESCs. The same workflow for identifying candidate enhancers was applied to other cell types and tissues.

Then, we counted the number of poly(A) RNA tags centred at candidate enhancers in −1 kb to 1 kb regions^1,33^, and we denoted the tag number of the i-*th* candidate enhancer in H1-hESCs by ${n}_{i}(i=1,2\ldots ,2,373)$. We clustered the candidate enhancers into two clusters based on $\mathrm{log}\,2({n}_{i}+1)$ using K-means clustering according to the method presented in a previous study^1^. A total of 837 enhancers belonged to a cluster with a median tag number of 123, whereas the other 1,536 enhancers were clustered into the other cluster with a median tag number of 4. We defined the 837 candidate enhancers with a significant level of transcription as eRNA regions and the other 1,537 candidate enhancers as weakly-transcribed enhancers. The minimal tag number of the enhancers classified as eRNA regions was 31, whereas the maximal tag number of the weakly-transcribed enhancers was 30; thus, the threshold number of poly(A) RNA tags for eRNA regions detection was 30. The eRNA regions in other cell types and tissues were detected with a corrected threshold, $30\times \frac{N}{245,647,806}$, where 30 was the threshold for eRNA regions detection in H1-hESCs, 245,647,806 was the total number of RNA tags in H1-hESC, and *N* was the total number of RNA tags in the other cell types or tissues. The corrected threshold alleviated the impact of sequencing depth in different cell types and tissues. For each cell type or tissue, the orientation of eRNA transcription was assessed on the basis of the direction given by the most RNA-seq tags.

### Characterization of eRNA regions

We estimated the extent of constraint on eRNA regions and weakly-transcribed enhancers by calculating the distribution of the PhastCons conservation scores in each region. Additionally, we used the genome structure correction (GSC) statistics^91^ to calculate confidence intervals for the degree of overlap between evolutionarily constrained bases in eRNA regions and in weakly-transcribed enhancers.

We used HOMER (http://homer.salk.edu/homer/interactions/)^92^ to process the Hi-C data in H1-hESCs^93^. We used the liftOver tool of the UCSC Genome Brower^94^ to transform the Hi-C data from reference NCBI36/hg18 to reference NCBI37/hg19. The resolution of the Hi-C data processed with HOMER was set to 1 kb, and ChromSDE^95^ was used to predict the chromatin 3D structures of enhancers.

We determined the enrichment of 466 TFs in eRNA regions and weakly-transcribed enhancers using FIMO^96^ with the default parameters. The TF motifs were collected from the Transfac^97^, Jaspar^98^, and UniProbe^99^ databases. A motif was retained only when it was significantly overrepresented (*P* ≤ 0.01) compared with the background sets. We used the binomial distribution test to calculate the *P*-values of TF enrichment for eRNA regions relative to the whole genome, and we used Fisher’s exact test to calculate the *P*-values of TF enrichment for eRNA regions relative to weakly-transcribed enhancers. Finally, we employed the standard normal distribution to transform the *P*-values to corresponding *z*-scores.

To assess the rate of discovery of new eRNA regions, we performed a saturation analysis on the element count and genomic coverage of enhancer RNAs using a Weibull distribution $({r}^{2}\ge 0.999)$, as described in our recent study^40^.

For GO analysis, a subset of 14 human datasets that represent the diversity of cell types and tissues were selected from the total 50 cell types and tissues. For each cell type/tissue, the genes that were associated with eRNA regions in that cell type/tissue and no more than three other cells/tissues in the subset were analysed using DAVID (http://david.abcc.ncifcrf.gov/home.jsp)^100,101^. For each cell type/tissue, the four top scoring categories were selected for display, and the threshold *P*-value for scoring as a top category was set to 10^−3^.

We generated the master list of all enhancers across the 14 cell types/tissues using our previous method^40^ and calculated the FPKM value for each eRNA region and its associated gene. Then, we calculated the Spearman correlation coefficient between the FPKM values of each eRNA and its associated gene.

### Detecting known and novel structural ncRNAs

To discover known structural ncRNAs in lymphoid eRNA regions, a sequence database was built by extracting the sequences of eRNA regions from 10 CD cells in the hg19 reference genome according to methods presented in previous studies^3,24^. We used “cmsearch” from the Infernal suite^43,44^ to search the 2,450 CM motifs derived from the Rfam database V12.0^45^ against the sequence databases using the default parameters. Hits with *e*-values < 1e-10 were collected. Hits for the same model in the same region or a region with a shift of a few bases across different cells were considered to be the same hit.

To detect novel structural ncRNAs in lymphoid eRNA regions, we first extracted the 100-way multiple alignments file of the eRNA regions in different cells from the stitched hg19 100-way multi-alignment file. Three programs, RNAz (version 2.1)^54^, REAPR (version 1.0)^22^, and EvoFold (version 1.0)^55^, were applied to detect ncRNAs using these genome-wide alignments. For RNAz and REAPR, we used scripts from RNAz to slice multi-alignment files into windows of size 120 and slide 40, and we filtered the spliced files using the default parameters. The filtered files were scored with RNAz and REAPR on both strands with the default parameters. Predictions with final *P*-values higher than 0.5 were retrieved for the subsequent pipeline. For the EvoFold^55^ analysis, sequences with > 20% gaps relative to the human genome were first removed. Then, alignments with sequences from fewer than six species were eliminated. EvoFold was then applied to the concatenated alignments and their reverse complements in 120 long overlapping windows, and each was offset by 40. Predictions with fewer than 10 pairing bases or an average stem length less than 3 and predictions that overlapped repeats or retrogenes were eliminated. Finally, the set was reduced to single coverage by removing the lowest-scoring candidates if overlap occurred, and the remaining candidates were ranked according to score.

Predictions detected by at least two of the aforementioned methods were retained for the subsequent analysis, and the corresponding predicted structures were selected first on the basis of the EvoFold rank and then by REAPR and RNAz. The predicted loci in the candidate set were identified by a local BLASTN^56^ search against the Rfam database using the default parameters. The whole predicted set was clustered by NoFold^57^ into the RNA empirical structure space (RESS). The top-ranked CMs were used to rank the most similar known structures to each novel structure in the cluster.

### Annotating newly identified lncRNAs

To annotate newly identified lncRNAs, we followed the pipeline used in a recent study^46^. Briefly, we first used the STAR aligner^102^ to align paired-end reads to the human genome by following a 2-pass alignment strategy^103^ with the parameter “–twopassMode Basic”. All the paired-end alignments overlapping at least one putative intron were retrieved and pooled. Second, filtered alignments collected from all samples were pooled and used as input for References Annotation Based Transcript (RABT)^104^, and the RefSeq human reference annotation was employed to optimize the performance of RABT^104^. To correct the transcript predictions derived from RABT, we filtered out the following set of transcript predictions: a) those with a total exonic sequence length less than 200 bp, b) predictions with FPKM = 0, and c) monoexonic predictions. Third, using cuffcompare, we compared our transcript predictions with all the genes from GENCODE with biotype annotations other than protein-coding or lncRNA^105^. We retained only those transcripts flagged as “novel” by cuffcompare. For the remaining transcripts, we calculated their coding potential using two methods, CPAT^60^ and CPC^61^, to exclude possible unannotated protein-coding or pseudogene loci. We ran CPAT on several non-coding gene sets using default parameters and the pre-defined threshold provided by the CPAT training procedure on human genes. Approximately 95% of our transcript predictions were predicted to be non-coding. Similarly, we computed CPC scores for our lncRNA candidates. In addition, the transcripts with open reading frame length > 300 were filtered from the results for both methods. Transcripts that did not show coding potential according to either CPAT or CPC were retained to generate a set of candidate lncRNAs. Fourth, the resulting set of candidate lncRNAs was used to build the final gene set and annotate novel lncRNAs as follows: candidate lncRNAs were merged with previous lncRNA annotations from both GENCODE V19^32^ and LNCipedia^62^ (V2.1) using cuffmerge^105^. The merged lncRNA annotation was further filtered to remove transcripts that had significant exonic overlap with GENCODE protein-coding genes. Specifically, we employed cuffcompare again and retained only those non-coding transcripts with class codes labelled “x”, “‘i”, “j”, “u”, and “o”.

At the gene level, the final annotation used throughout the manuscript includes 20,345 protein-coding genes from GENCODE and 18,268 non-coding genes. A gene transfer format (gtf) file representing this reference annotation was used for all downstream analysis. A gtf annotation file for the non-coding genes is available as Table S6. The non-coding genes include 3,880 novel genes (defined as genes with no overlap with the ones in the GENCODE^32^ or LNCipedia^62^ databases). A total of 80% of the non-coding genes (14,629) were classified as fully intergenic. A total of 4,654 non-coding loci generated at least one novel isoform. Finally, a total of 3,359 non-coding transcripts that overlap with the eRNA region master list were then extracted from the final annotated results for the downstream analysis.

### Prediction of SNP effects on structural ncRNAs

We used RNAsnp (version 1.1)^26^ to predict the effects of SNPs on the RNA secondary structure of structural ncRNAs using the default parameters with mode 1 for short RNA sequences (<1,000 nt) and with mode 2 for large RNA sequences. For each autoimmune disease-associated SNP collected from the dbSNP^106^, GWAS SNP^65^, and ClinVar^107^ databases, RNAsnp considered a window of + /−200 nt around the SNP position to generate the wild-type (WT) and mutant (MT) subsequences and compute their respective base pair probability matrices. Then, the structural difference between WT and MT RNA sequences was calculated on the basis of Euclidean distance for all sequence intervals within the subsequence. Finally, a local region predicted with the maximum Euclidean distance and the corresponding *P*-value was reported. The riboSNitches were predicted to be those RNAs showing large structural disruptions caused by autoimmune disease-associated SNPs according to RNAsnp with *P*-values < 0.2, and they are shown in Table S7.

### Availability

The enhancer RNAs identified across 50 human cell/tissue types have been deposited with the Gene Expression Omnibus under the accession ID GSE99453.

## Electronic supplementary material

Supplementary Material

Supplementary Table1

Supplementary Table2

Supplementary Table3

Supplementary Table4

Supplementary Table5

Supplementary Table6

Supplementary Table7
