# Supplementary material for: Functional annotation of structural ncRNAs within enhancer RNAs in the human genome: implications for human disease
Source: Sci Rep. 2017 Nov 14;7:15518. doi: 10.1038/s41598-017-15822-7 (PMC5686184; doi:10.1038/s41598-017-15822-7)

Supplementary materials for

Functional annotation of structural ncRNAs within enhancer RNAs in the human genome: implications for human disease

Chao Ren[1¶], Feng Liu[1,2¶], Zhangyi Ouyang[1], Gaole An[1], Chenghui Zhao[1], Jun Shuai[2], Shuhong Cai[2], Xiaochen Bo[1*], Wenjie Shu[1*]

[1]Department of Biotechnology, Beijing Institute of Radiation Medicine, Beijing, China

[2]Department of Information, The 188th Hospital of ChaoZhou, ChaoZhou, China

[*]Corresponding author

E-mail: shuwj@bmi.ac.cn (WS)

E-mail: boxc@bmi.ac.cn (XB)

[¶]These authors contributed equally to this work.

Supplementary Figures

Figure S1. Workflow for identifying intergenic candidate enhancers in hESCs

Figure S2. UCSC browser display of 12 multi-omic signatures at candidate enhancers in hESCs, including 6 histone modifications, 2 transcription factors and 1 cofactor, RNA polymerase II, DHSs, and DNA methylation

Figure S3. Twelve multi-omic signature of candidate enhancers, promoters, lincRNA and rRNA genes in hESCs, including 6 histone modifications, 2 transcription factors and 1 cofactor, RNA polymerase II, DHSs, and DNA methylation

Figure S4. Characterization of eRNA regions and weakly-transcribedenhancers in hESCs

(A) The distribution of domain sizes of eRNA regions (left) and weakly-transcribed enhancers (right) in hESCs.

(B) The distribution of distance from the centres of eRNA regions (red) and weakly-transcribed enhancers (blue) to their nearest transcription start site (TSS).

(C) The distribution of the evolutionary conservation of eRNA regions (red) and weakly-transcribed enhancers (blue).

(D) The 3D chromatin structure predicted around eRNA regions (up) and weakly-transcribed enhancers (down).

Figure S5. Association between the expression levels of eRNAs and their associated genes across 14 human cell and tissue types

(A) Heatmap showing the classification of eRNA domains across 14 human cell and tissue types. Colour scale reflects the density of RNA-seq signal at the eRNA regions. (B) Heatmap showing the classification of eRNA-associated genes across 14 human cell and tissue types. Colour scale reflects the density of RNA-seq signal at the eRNA-associated genes.

Figure S6. Eight multi-omic signatures of enhancers identified by two methods in hESCs, including 3 histone modifications, 2 transcription factors and 1 cofactor, RNA polymerase II, and DHSs

Figure S7. Eight multi-omic signatures of eRNA regions and e-lncRNA in hESCs, including 3 histone modifications, 2 transcription factors and 1 cofactor, RNA polymerase II, and DHSs

Figure S8. Detection of riboSNitches in lymphoid eRNAs

(A) Distribution of the effect of SNPs on ncRNAs in lymphoid eRNA regions and

random loci. RNAsnp was used with default parameters to predict the effect of SNPs on local RNA secondary structure.

(B) The partial effect of SNP rs19942260 on TERC in lymphoid eRNA regions.

(C) The partial effect of SNP rs2038013 on TRAF3IP2-AS1 in lymphoid eRNA regions.

(D) The partial effect of SNP rs11950065 on RP11-175K6.1-001 in lymphoid eRNA regions.

(E) The partial effect of SNP rs11762252 on lnc-TSPAN33-2:1 in lymphoid eRNA regions.

Supplementary Tables

Table S1. Right-tailed P-value of TF binding motifs in eRNA regions compared to 3 types of background (whole genome, promoter, and CpG islands).

Table S2. Transcription factor (TF) binding motifs enriched at eRNA regions with P-value<1e-5 compared with 3 types of background (whole genome, promoter, and CpG islands).

Table S3. Log2 ratio and P-value of TF binding motif enrichment within eRNA regions relative to weakly-transcribed enhancers.

Table S4. Comparison of enhancers identified by our pipeline and Roadmap Epigenome Consortium.

Table S5. Public sequencing datasets used in this study.

Table S6. Novel lncRNA transcript discovered in CD cells in gtf format.

Table S7. Assessment of riboSNitch effects on lncRNA.

Supplementary Figure. 1

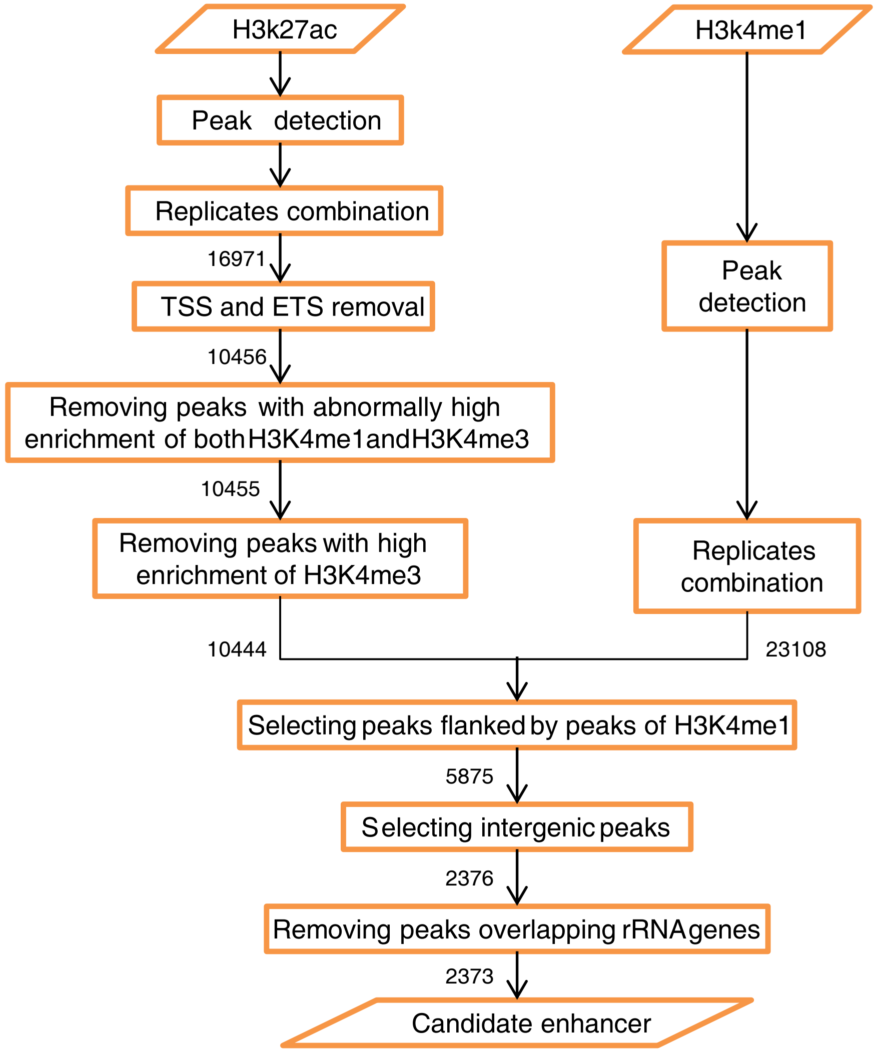

Supplementary Figure. 2

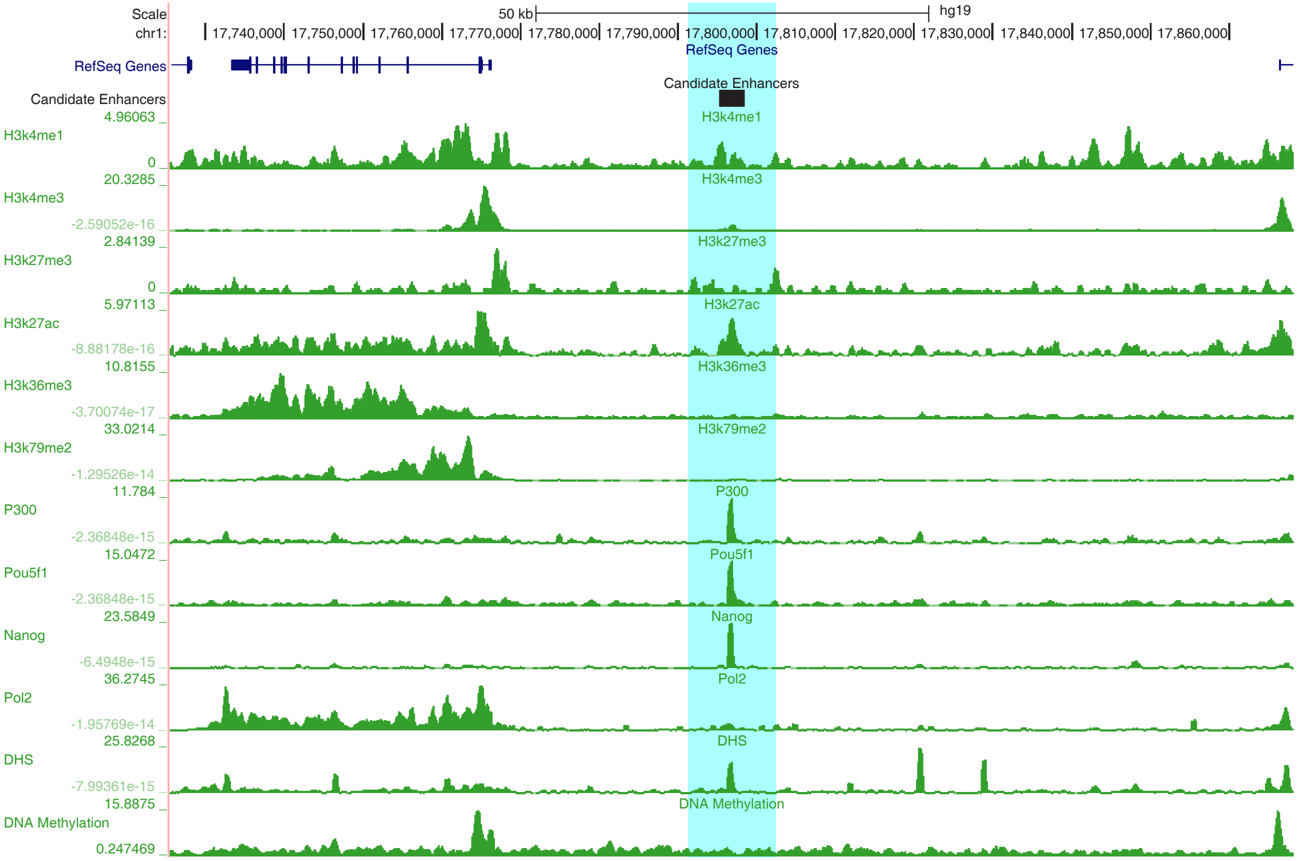

Supplementary Figure. 3

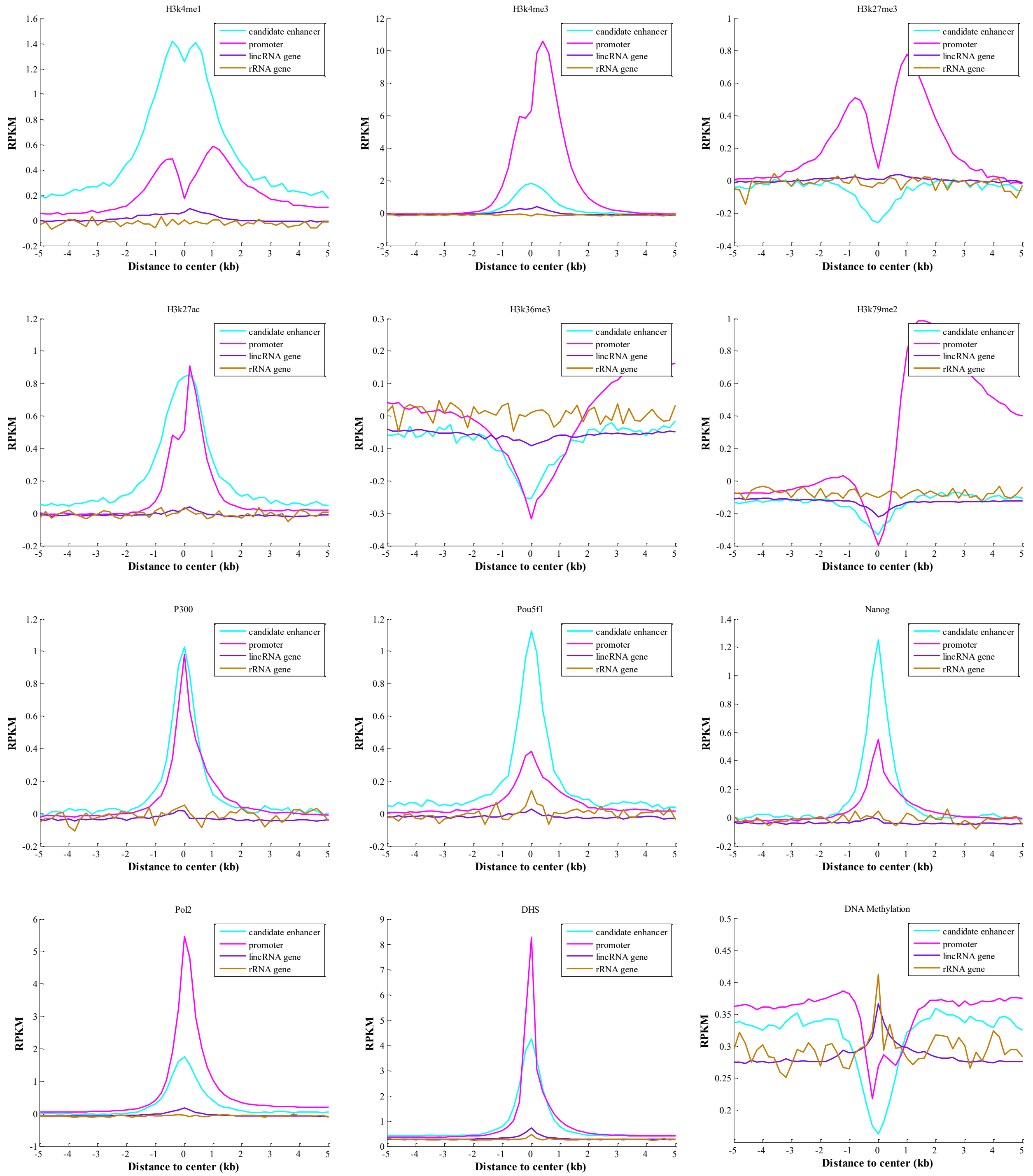

Supplementary Figure. 4

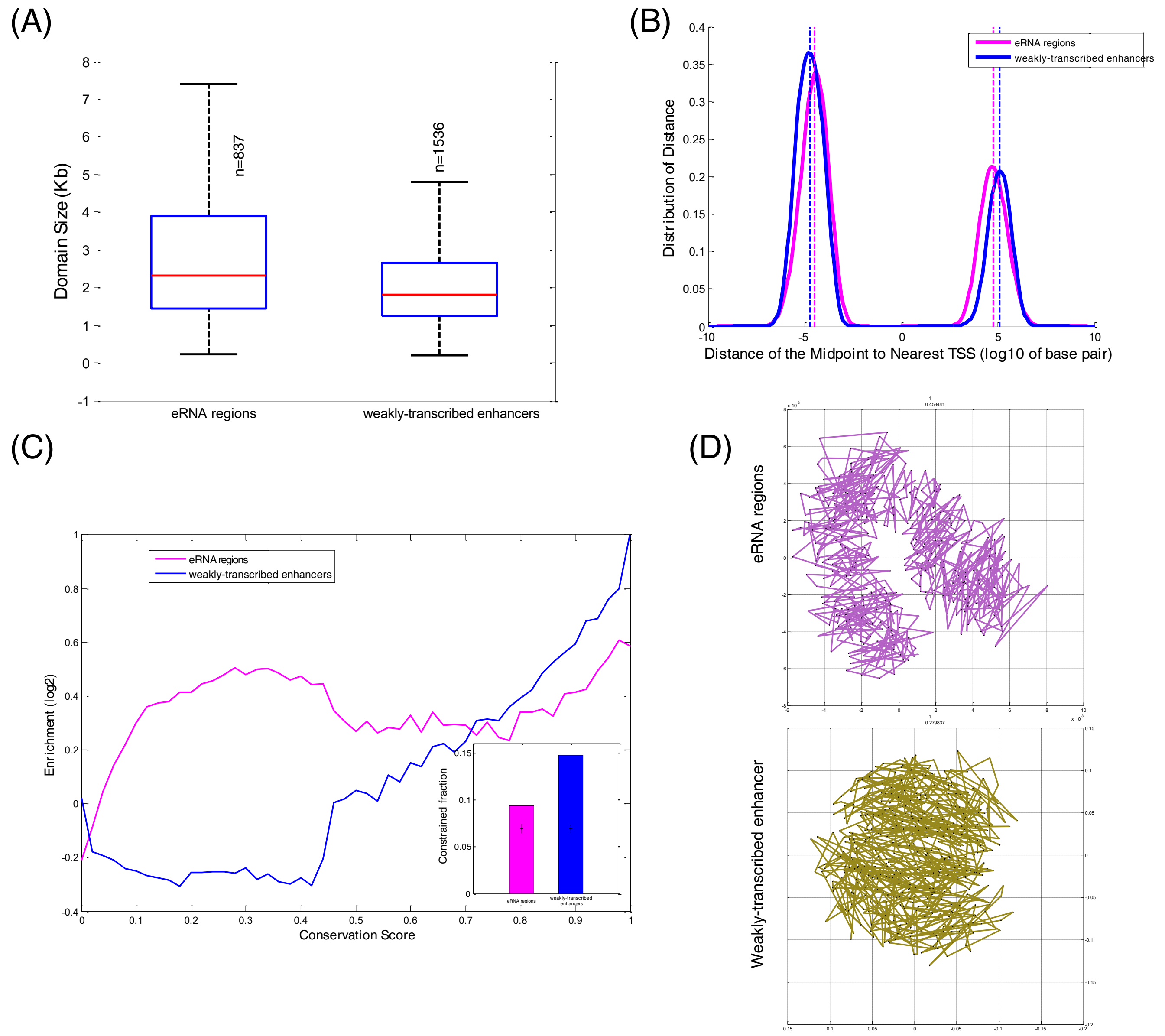

Supplementary Figure. 5

(A)

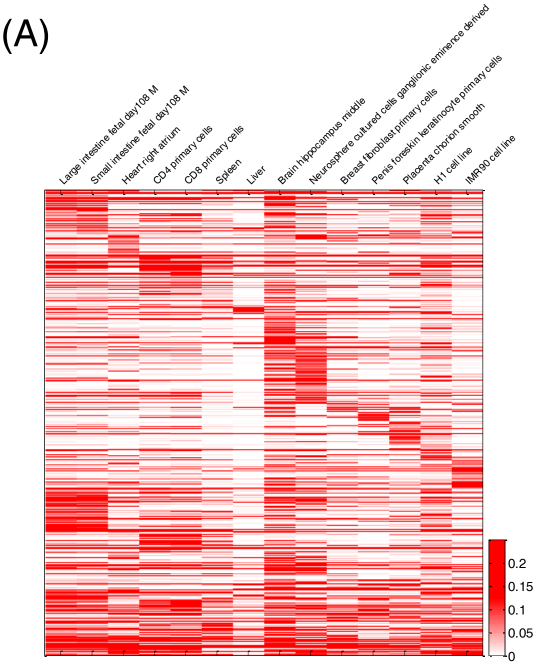

eRNA level

(B)

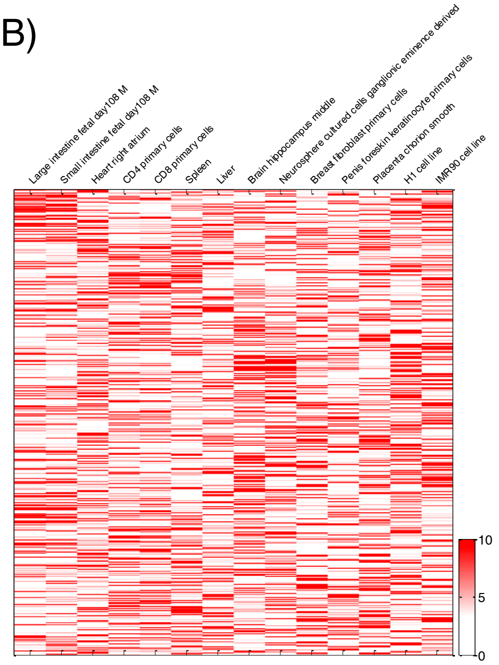

mRNA level of associated genes

Supplementary Figure. 6

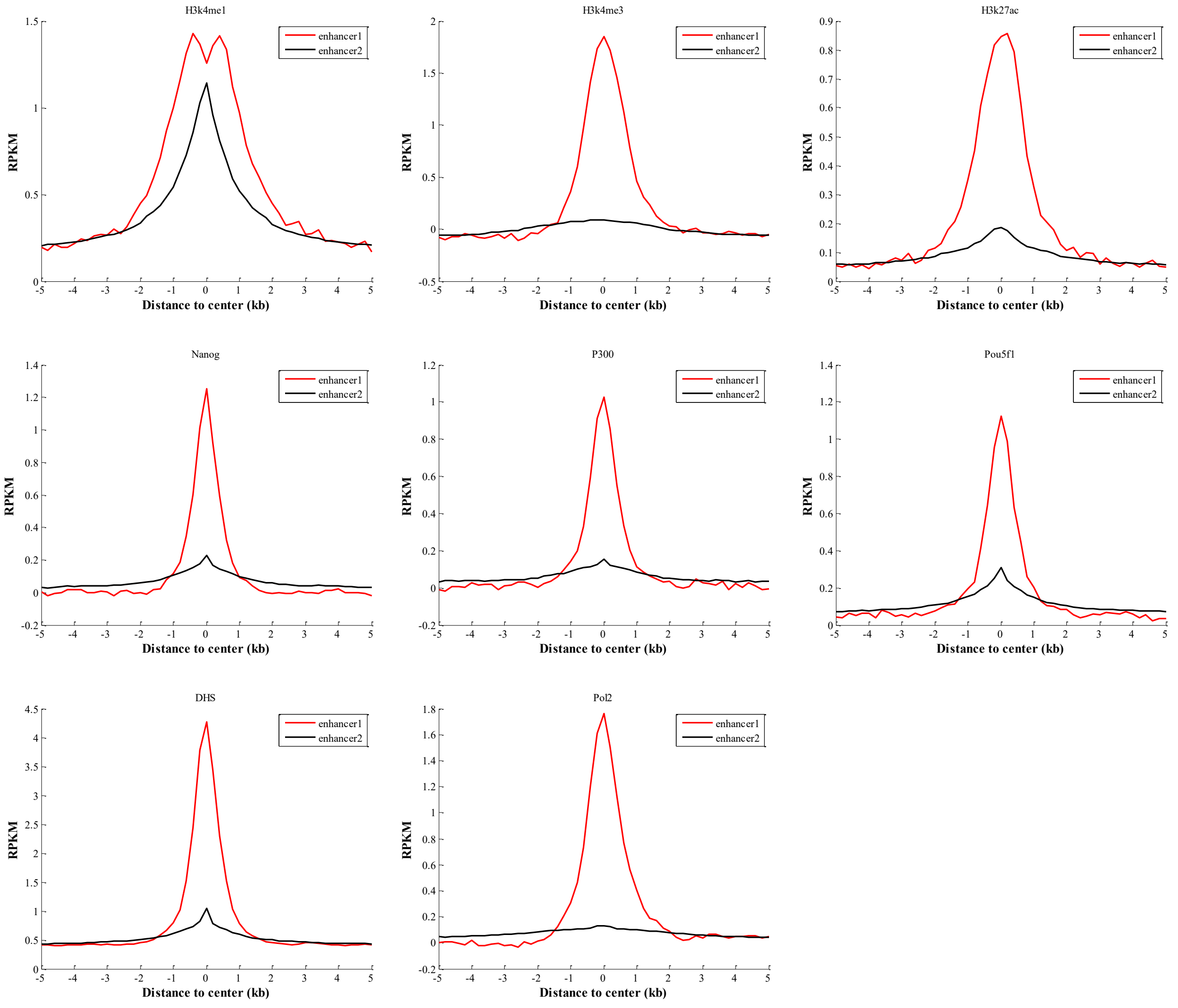

Supplementary Figure. 7

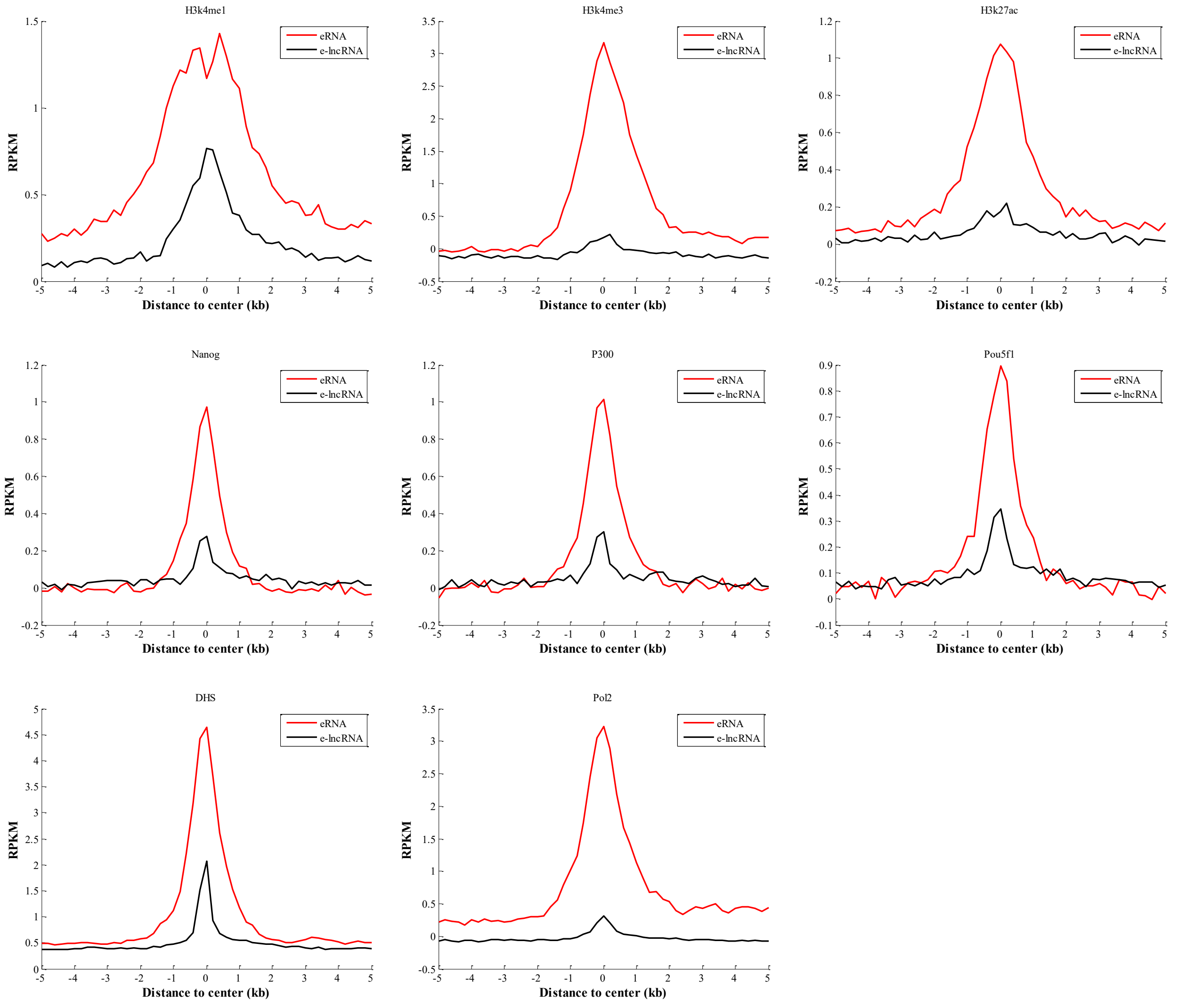

Supplementary Figure. 8

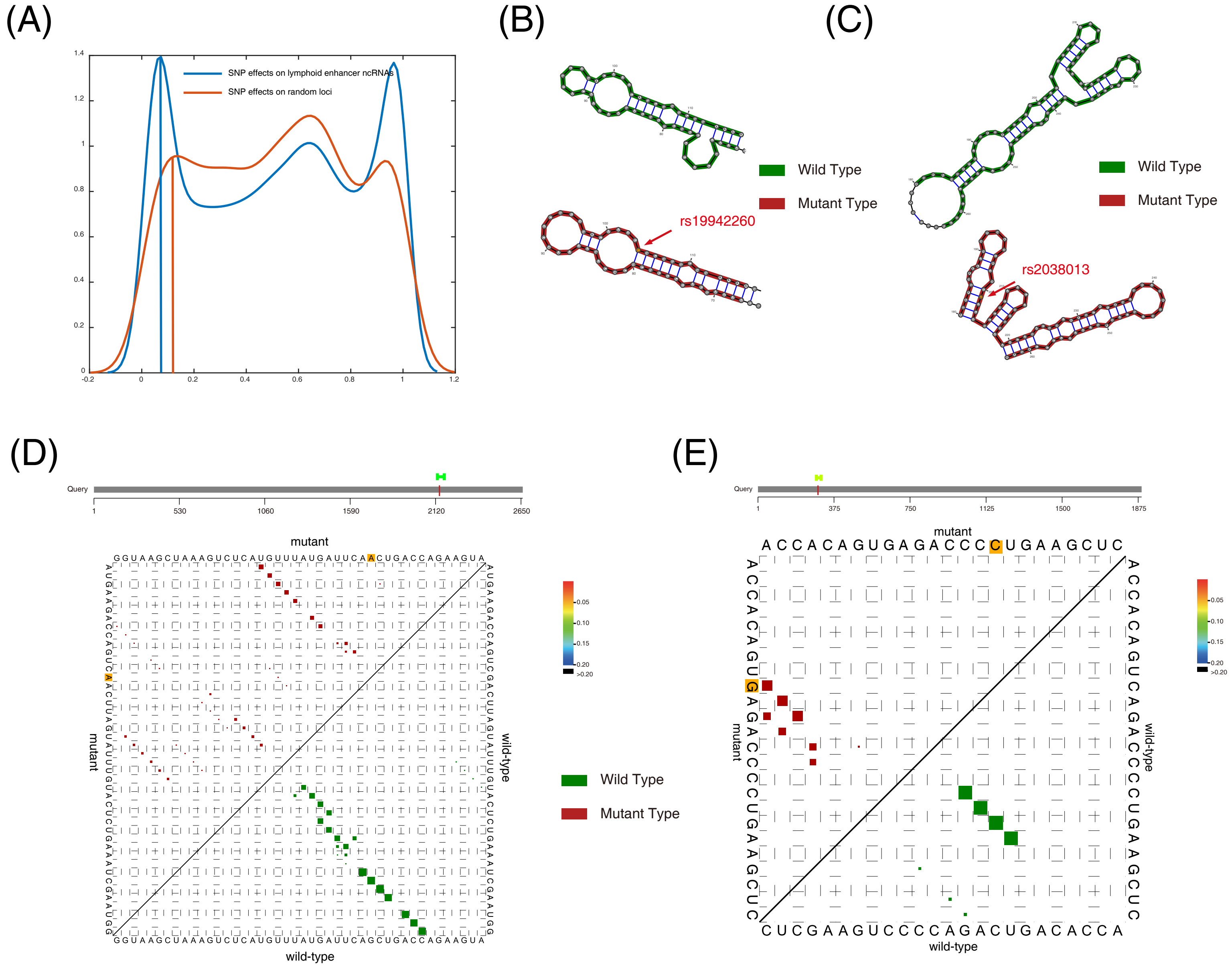

Supplement: Supplementary file 1 — Supplementary Material [file 41598_2017_15822_MOESM1_ESM.pdf]
